# Elevated level of beta-D-glucan in *Pseudomonas* infection

**DOI:** 10.1016/j.idcr.2024.e01931

**Published:** 2024-02-13

**Authors:** K. Shrestha, K. Kadkhoda

**Affiliations:** a Infectious diseases division, Akron General, Cleveland Clinic, Cleveland, OH, USA; b Immunopathology Laboratory, Laboratory Medicine Division, Diagnostics Institute, Cleveland Clinic, Cleveland, OH, USA.; c Cleveland Clinic Lerner College of Medicine, Case Western Reserve University

**Keywords:** Beta-D-Glucan, Fungitell, *Pseudomonas*

## The case

A 67-year-old woman with deafness and mutism visited Akron General Medical Center in May 2023, complaining of worsening dyspnea, productive cough, and pleuritic chest pain for three days. Before admission, she had been experiencing breathlessness for almost a month and had been prescribed oral steroids and azithromycin, which did not improve her condition. The patient's medical history included chronic obstructive pulmonary disease, chronic hypoxic respiratory failure, rheumatoid arthritis treated with Golimumab and methotrexate, osteoporosis, peptic ulcer disease, latent tuberculosis infection (treated previously), lung nodules, prior venous thrombosis, and Takotsubo cardiomyopathy.

Due to her hypoxic condition, she required noninvasive positive pressure ventilatory support. Her complete blood count showed leukocytosis with a white blood cell count of 24,700 /µL (reference range: 3700–11,000), and the nasopharyngeal swab tested positive for Rhinovirus/Enterovirus nucleic acid. The chest x-ray did not indicate dense consolidation. Given her immunosuppressed condition and subacute dyspnea, an extensive workup was conducted for opportunistic infections, including fungal infections. Her sputum culture grew a few hypermucoid colonies of *Pseudomonas aeruginosa*, and serum 1,3-β-D-Glucan (BDG) (Fungitell®; Associates of Cape cod Inc.) was positive at 301 pg/mL (reference range: <60). Serum *Coccidioides spp*. IgG antibody test was low positive (index value: 1.6; positivity cutoff: 1.5), while the remaining fungal workup was negative. The blood lactate dehydrogenase level was normal, and the sputum tested negative for *Pneumocystis jirovecii* by polymerase chain reaction. After treatment with broad-spectrum antibiotics followed by de-escalation to cefepime to complete the course, the patient's respiratory status improved to her baseline. The patient had never left Ohio which is not endemic for *Coccidioides*. Due to her low likelihood of having *Coccidioides* infection, we concluded that the positive *Coccidioides* IgG result was false positive. This could be due to her high rheumatoid factor level that she had in the past during her rheumatoid arthritis work-up. We considered whether the *Pseudomonas* infection could have caused a positive result in the BDG assay.

## Discussion

BDG, a type of polysaccharides found in the cell wall of most fungi, has proven to be a valuable diagnostic target for detecting invasive fungal infections, particularly in individuals with compromised immune system. Some fungi that yield positive BDG results include yeasts such as *Candida*, *Trichosporon*, and *Saccharomyces*, molds like *Acremonium*, *Aspergillus*, and *Fusarium*, as well as dimorphic fungi such as *Coccidioides*, *Histoplasma*, and *Sporothrix*, and others like *Pneumocystis* [1]. However, it is important to note that certain fungi, such as *Cryptococcus*, *Blastomyces*, and zygomycetes, produce only small to undetectable amounts of BDG, thereby reducing its reliability in diagnosing infections caused by such fungi. A few tests are available to measure the levels of BDG in the blood, all of them follow the *Limulus* Amoebocyte Assay principle. One such test is the United States Food and Drug Administration-approved Fungitell® assay. A serum level exceeding the cutoff value of 80 pg/mL is deemed significant. Given the challenges associated with diagnosing invasive fungal diseases and the associated high morbidity and mortality, this culture-independent diagnostic tool can significantly enhance clinical outcomes, particularly in critically ill patients where obtaining tissue samples can be problematic and fungal cultures may not always be sensitive enough [2]. The sensitivity and specificity of serum BDG testing for diagnosing invasive fungal infection vary depending on the cutoff values, study sample size, and other confounding factors. The sensitivity and specificity have ranged from 57% to 97%, and from 56 to 93%, respectively [1].

Ostrosky-Zeichner *et al*. published their findings from their study performed in a multicenter setting for evaluating the BDG assay as an aid in diagnosing fungal infections. They demonstrated that using the assay resulted in high specificity and positive predictive value and was a useful diagnostic adjunct for invasive fungal infections. When the cutoff value for the assay was set at 80 pg/mL, the sensitivity and specificity were 64.4% and 92.4%, respectively. The positive predictive value was 89%, and the negative predictive value was 73% [2]. Senn *et al*. showed that monitoring with BDG assay (by Wako colorimetric method) is a proper non-invasive method for early diagnosis of invasive fungal infections in patients with acute leukemia. They found that the time interval between the onset of fever as the first sign of invasive fungal infection and BDG assay positivity was significantly shorter than the time to diagnosis of invasive fungal infection by clinical, microbiological, radiological, and histopathological criteria [3]. Specificity was excellent at 96%. All false positive assay results in the remaining 4% were observed in patients with gastrointestinal fungal colonization and/or mucositis. Another study had shown relatively high rates of false positive in BDG assay [4] in which they tested blood samples from healthy blood donors, patients with blood cultures positive for yeasts or bacteria, and those with positive *Aspergillus* galactomannan or *Histoplasma* antigen. Twenty-two of 36 samples from 15 patients with blood cultures positive for gram-positive cocci were BDG-positive. One of those patients had a positive biopsy for *Candida* as well, and it was a true positive. One patient received kidney dialysis. One patient received intravenous immunoglobulin. Five of them received different beta-lactam antibiotics. Of the ten patients with blood cultures positive for gram-negative bacilli, three had more than one specimen positive for BDG. Two had at least one sample indeterminate. Out of 3 positives, one patient had received intravenous immunoglobulin and a beta-lactam antibiotic. One had received no antibiotics, and the other had only received doxycycline. Three other patients with gram-negative bacilli had received beta-lactam antibiotics but were BDG-negative. The sensitivity and specificity of the BDG assay in the study sample, including bacteremic patients, were 93.3% and 77.2%, respectively. The positive predictive value was only 51.9%. The negative predictive value was more than 95%.

Several factors can cause the so-called false positive BDG assay results, as reported by various authors [1], [2], [3], [4], [5], [6], [7], [8], [9]. These factors include patients undergoing hemodialysis, those receiving intravenous immunoglobulins or blood products that contain high levels of BDG, such as albumin and coagulation factors, and those with open wounds exposed to gauze or other materials that have BDG. Additionally, some streptococci, such as *S. mitis*, and *Alcaligenes faecalis*, can produce BDG [5]. Exposure to anti-tumor polysaccharides and antibiotics derived from fungal sources such as beta-lactams can also cause false positives. Another potential cause is mucosal damage from chemotherapy or radiotherapy. By breaching the mucosal barrier, mucositis allows dietary glucan or colonizing *Candida* to enter the bloodstream usually at low levels. One of the other bacteria that can produce BDG is *Pseudomonas*. Yang *et al*. purified polysaccharides from soil samples and observed that the highest reported yield of curdlan, a polysaccharide consisting of beta–1, 3–linked glucose residues, was produced amply by *Pseudomonas* species [10].

The findings reported by Mennink-Kersten *et al*. highlight *Pseudomonas aeruginosa* as a cause of BDG assay positivity [8]. They tested the BDG reactivity from culture supernatants of different bacteria commonly found in the blood of immunocompromised patients. They found that in addition to *Alcaligenes* and *Pneumococcus*, *Pseudomonas aeruginosa* showed BDG reactivity. Two of nine hematology patients with *Pseudomonas* bacteremia had serum BDG reactivity higher than 80 pg/mL cutoff value on the same day the blood cultures were drawn. The reactivity in the supernatant of all seven remaining cultures decreased after treatment with the enzyme 1,3-beta-D-glucanase, suggesting the specificity of the reaction. These patients were not receiving any glucan-containing antibiotics and were ruled out for invasive fungal disease. Most other clinical isolates tested, including gram-negative pathogens such as *E. coli*, *Proteus mirabilis*, and *Salmonella*, showed no significant reactivity. Koya *et al*. analyzed the reactivity of BDG assay with *Pseudomonas aeruginosa* bacteremia using another testing method (beta-glucan Test Wako)[7]. Their study reviewed the BDG assays of 47 patients with *P. aeruginosa* bacteremia who did not have evidence of fungal infections at the University of Tokyo Hospital between 2003 and 2011. The results showed that BDG reactivity was detected in 6 (12.8%) patients based on the reference cutoff value of less than 11 pg/mL. Interestingly, they also found that the levels of BDG and procalcitonin increased simultaneously in cases of *P. aeruginosa* bacteremia. However, there was some time lag between the elevation of BDG and procalcitonin levels in patients with invasive fungal infection.

Here, we need to highlight that the word “false” in false positive, may be misleading as the G factor present in horseshoe crab from which the BDG assay reagents are prepared, naturally binds BDG to start the functional cascade. BDG is not just a binding assay but rather a functional kinetic one (a bioassay). If we simply base our benchmark on detecting fungi like *Candida*, then any other positive BDG result might be labelled as “false” but in reality the result is not “analytically false”, thus we rather need to tease out the source that has produced this analyte, be it fungi, bacteria, or a dialysis membrane for instance.

All in all, given the clinical picture that we described earlier for our patient and the discussion, we could not find any other reason for such high elevation in her BDG level. She fully recovered on anti-*Pseudomonas* agents; therefore, we believe the most likely cause for her serum BDG elevation was her concomitant *Pseudomonas* infection. Given the hypermucoid nature of the colonies, it is reasonable to assume that this strain produced high amount of curdlan and BDG as a result, which was released in the tissue environment and absorbed into circulation. More studies are needed to substantiate this finding in a large group of patients.

## Conflict of interest statement

None declared.
